# Percutaneous ethanol injection *versus* conservative treatment for benign cystic and mixed thyroid nodules

**DOI:** 10.1590/2359-3997000000120

**Published:** 2016-02-11

**Authors:** Mari Cassol Ferreira, Camila Piaia, Ana Carolina Cadore

**Affiliations:** 1 Faculdade de Medicina da Universidade de São Paulo Laboratório de Carboidratos LIM-18 Faculdade de Medicina da Universidade de São Paulo (FMUSP), Laboratório de Carboidratos LIM-18;; Universidade Comunitária da Região de Chapecó Chapecó SC Brasil Universidade Comunitária da Região de Chapecó (Unochapecó), Chapecó, SC, Brasil; 2 Universidade Comunitária da Região de Chapecó Chapecó SC Brasil Universidade Comunitária da Região de Chapecó (Unochapecó), Chapecó, SC, Brasil

**Keywords:** Thyroid nodule, etanol, interventional ultrasonography, percutaneous administration, ultrasonography

## Abstract

**Objective:**

: To evaluate the efficacy and safety of percutaneous ethanol injection (PEI) in reducing the volume of cystic and mixed thyroid nodules.

**Materials and methods:**

: A total of 36 patients with nodules treated with PEI and 13 individuals who declined PEI and were followed clinically or received other non surgical treatment (control group). Assessments were performed at baseline (immediately before treatment in the PEI group or evaluation of the nodule on ultrasonography in the control group) at short-term (on average 30 days after the last injection in the PEI group), and long-term (on average 14 months after baseline in the PEI group or 26 months after baseline in the control group).

**Results:**

: In the PEI group, the mean baseline volume of 10.4 ± 9.8 cm^3^ reduced at short-term follow-up to 2.9 ± 3.1 cm^3^ (67.7 ± 19.9%, p < 0.001) and at long-term follow-up to 2.0 ± 2.5 cm^3^ (78.2 ± 19.5%, p < 0.01 *versus* baseline and p = 0.009 *versus* short-term follow-up). Both types of nodules showed similar degrees of reduction. In the control group, mean volume was 5.8 ± 3.4 cm^3^ at baseline and 6.2 ± 3.0 cm^3^ at long-term follow-up (p = 0.507). Compared with the control group, the PEI group showed larger reduction (p < 0.001).

**Conclusions:**

: PEI is effective in reducing the volume of cystic and mixed benign thyroid nodules, with sustained long-term efficacy and better outcome when compared with conservative therapies. Treatment with PEI is a safe alternative, with minimal, transient and self-limited adverse events.

## INTRODUCTION

Thyroid nodules are characterized by excessive structural growth, functional transformation, and/or cystic degeneration of one or several areas within the gland (1). These nodules are detected in 4 to 8% of the adult population when evaluated by palpation, and between 13 to 67% when evaluated by ultrasonography (2).

The progression and management of thyroid nodules are still controversial. Conventional and conservative treatment modalities have shown unsatisfactory results in reducing the volume of the nodules and raised concern about possible side effects (3,4). Surgery is a long-established therapeutic option for benign thyroid nodules. However, the cost of thyroid surgery, risk of temporary or permanent complications and impact on quality of life remain relevant concerns. Therefore, attempts have been made during the past two decades to conduct minimally invasive treatments without general anesthesia, and with minimal damage to the skin and cervical structures (5,6).

Percutaneous ethanol injection (PEI) was originally introduced in the 1980’s as a therapeutic option for renal cysts. In 1989, PEI was proposed as a treatment for benign cystic thyroid nodules (7). In 1990, PEI was shown to improve hyperthyroidism and reduce the size of hyperfunctioning nodules (8). Intranodular injection of ethanol leads to a complex and irreversible local injury associated with hemorrhagic infarction, thrombus formation, coagulative necrosis and fibrosis sparing areas outside the nodule (9). Therefore, PEI has been an increasingly common therapeutic alternative for thyroid nodules, with positive results confirmed by several groups within the last decade (10-14). Even though the efficacy and safety of PEI has been demonstrated in various studies, further evidence is still required to support its routine use in clinical practice. In particular, more evidence is still necessary about the efficacy of PEI compared with other treatments for thyroid nodules, as well as the effects of ethanol on different types of nodules. Based on that, we conducted a study to evaluate the efficacy and safety of PEI compared with conservative treatment in cystic and mixed thyroid nodules.

## MATERIALS AND METHODS

We selected 49 patients with thyroid nodules who attended a reference center for community care between 2006 and 2012 and were not previously treated with radioiodine, surgery, suppressive LT4 or PEI. In 36 patients, ultrasonography-guided PEI was recommended for treatment of a single or dominant nodule due to aesthetic and/or compressive complaints. Before treatment, all nodules were evaluated with fine needle aspiration biopsy and cytological analysis to exclude malignancy. Thirteen individuals who declined PEI and were only followed clinically or received another nonsurgical treatment were used as the control group. In the control group throughout the follow-up, 4 patients used suppressive doses of levothyroxine (L-T4); others were followed in clinical follow-up with expectant management. There were no cases with surgical treatment, and no clinical complaints in this group during the average period of 26 months of follow up.

Patients were eligible if aged ≥ 18 years and presenting one or more cystic or mixed nodules > 2.0 cm in the largest diameter or with volume > 1.0 cm^3^, non hyperfunctioning. One patient with a nodule smaller than 1 cm^3^ was included due to aesthetical discomfort caused by the position of the nodule. Exclusion criteria were pregnancy, nodules with malignant or suspicious cytology and solid nodules without cystic components. Treatment consisted of intranodular injection of sterile 99% ethanol guided by ultrasonography. The mean interval between sessions was 30 days and each patient received one or more injections according to their individual response to treatment. The amount of ethanol injected at each session corresponded to 30% of the volume of the nodule, with up to 3 mL per session. In patients with cystic or predominantly cystic nodules, we first aspirated the fluid component of the nodule before injecting the ethanol. We performed new ultrasonographic evaluations before each new session to measure the volume of the nodule and evaluate whether additional PEI would be necessary. Anesthesia was not required before the injections.

The follow-up of the cohort was divided into three periods: *baseline*, comprising the evaluation immediately before treatment in the PEI group or evaluation of the nodule on ultrasonography in the control group; *short-term follow-up*, 30 days after the last injection (PEI group); and *long-term follow-up*, on average 12 months after baseline (PEI group) or more than 12 months after baseline (control group). We defined a longer follow-up in the control group to avoid a short period of observation.

Based on ultrasonographic features, the nodules were subdivided into three groups: *cystic*, for nodules without solid parts; *predominantly cystic*, for nodules with fluid occupying ≥ 50% of the volume of the nodule; and *predominantly solid*, for nodules with fluid occupying < 50% of the total nodular volume.

The volume of the nodule was calculated using the ellipsoid formula and represented in cm^3^: anterior-posterior diameter (cm) X transversal diameter (cm) X longitudinal diameter (cm) X 0.52. To calculate the rate of nodular reduction after treatment, we applied the following formula: (initial volume – final volume) X 100/initial volume.

According to the decrease in nodular volume, PEI results were categorized as: *incomplete response* (if < 70% decrease) or *complete response* (if ≥ 70% decrease). Thyroid function was evaluated with serum levels of TSH and free T4 at baseline in both groups, at short-term follow-up in the PEI group and at long-term follow-up in the control group. All tests were processed in the same laboratory. Based on reference values, levels of TSH between 0.3 and 4.5 mIU/mL and free T4 between 0.8 and 1.4 ng/dL were considered normal.

The study was performed according to the Declaration of Helsinki after approval by the Ethics Committee for Research Involving Human Subjects of *Universidade Comunitária da Região de Chapecó*. All patients signed a free and informed consent form.

### Statistical analysis

The comparison of parametric quantitative variables between both groups was analyzed with Student’s t-test and non-parametric variables with the Mann-Whitney test. Qualitative variables were evaluated with the chi- square test. The comparison of volumes at different periods was conducted with the Friedman test, confirmed with nonparametric multiple comparison for dependent data. The same comparison in the control group was conducted with the paired Wilcoxon test. We applied the likelihood ratio to compare complete or incomplete response rates among different types of nodules. We then grouped cystic and predominantly cystic nodules to compare the outcomes with those with predominantly solid nodules using the chi-square test followed by confirmation with the generalized estimating equation using marginal binomial distribution with logit link function. The Spearman correlation test assessed the rate of volumetric decrease with clinical variables during follow-up. Multivariate analysis was performed with the Statistical Package for Social Sciences (SPSS^®^), version 19.0. We used a confidence interval of 95% and a significance level of 5%.

## RESULTS

Of 36 nodules treated with PEI, 13 were cystic, eight were predominantly cystic and 15 were predominantly solid. In the control group, three nodules were cystic, one was predominantly cystic and nine were predominantly solid. Three patients treated with PEI had baseline serum TSH < 0.3 mIU/mL.

Mean follow-up in the PEI group was 14.0 ± 9.4 months and in the control group was 26.0 ± 17.2 months (p = 0.06). Table 1 presents the clinical features of the patients in both groups. We performed 107 injections of ethanol in the PEI group, with an average of 2.9 ± 1.6 sessions per individual (median of 3 sessions). The volume of ethanol injected depended on the number of sessions and ranged from 0.4 mL to 15.5 mL, with an average of 4.4 ± 3.7 mL per nodule. The amount of fluid aspirated from the nodules ranged from 0 to 35 mL, with an average of 6.3 ± 9.0 mL. Table 2 presents a detailed description of the procedures according to the type of nodule.

**Table 1:** Clinical characteristics of patients in the PEI and control groups

	Treated (n = 36)	Control (n = 13)	p
Gender	34F/2M	13F	
Age (years)	40.4 ± 12.9*	47 ± 9.5*	0.09
Follow-up (months)	14 ± 9.4*	26 ± 17.2*	0.06
Type of nodule	13	3	0.21
Cystic	8	1	
Predominantly cystic			
Predominantly solid	15	9	
Baseline volume (cm^3^)	10.4 ± 9.8*	6.2 ± 3.1*	0.02

PEI: percutaneous ethanol injection; F: female; M: male.

* Mean ± standard deviation.

**Table 2:** Characteristics of the cohort, and description of follow-up, procedures and volumes according to the type of nodule

	Cystic (n = 13)	Predominantly cystic (n = 8)	Predominantly solid (n = 15)	Total
Mean age ± SD (years)	44.5 ± 12.9	38.4 ± 12.8	37.7 ± 12.8	40.4 ± 12.9
Follow-up (months)	18.9 ± 13.5	10.9 ± 4.6	11.5 ± 4.2	14.0 ± 9.4
Number of injections	2.7 ± 1.8	3.0 ± 1.1	3.1 ± 1.6	2.9 ± 1.6
Total volume of ethanol injected (mL)	4.5 ± 4.6	3.9 ± 3.3	4.6 ± 3.2	4.4 ± 3.7
Baseline volume (cm^3^)	11.6 ± 11.4	11.6 ± 11.1	8.8 ± 8	10.4 ± 9.8

SD: standard deviation.

At baseline, the volume of the nodules in the PEI group ranged from 0.7 to 40.0 cm^3^, with an average of 10.4 ± 9.8 cm^3^ (95% confidence interval [95% CI] 7.1–13.7 cm^3^). At short-term follow-up, the volume of the nodules ranged from 0.01 to 13.30 cm^3^, with an average of 2.90 ± 3.10 cm^3^ (95% CI 1.80–3.90 cm^3^), reflecting a reduction of 67.7 ± 19.9% compared with baseline (p < 0.001). At long-term follow-up (average of 14 months), the nodules ranged in volume from 0.01 to 9.80 cm^3^, with an average of 2.0 ± 2.5 cm^3^ (95% CI 1.2–2.9) reflecting a reduction of 78.2 ± 19.5% when compared with baseline (p < 0.001). Compared with the volume at short-term follow-up, the reduction at long-term follow-up showed a significant difference (p = 0.009).

**Figure 1:**
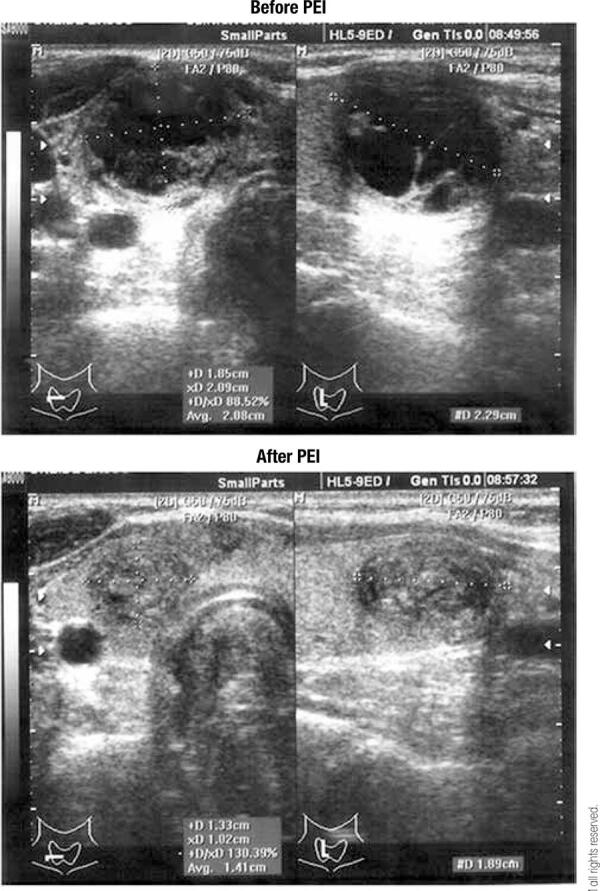
Sequence of ultrasonographic images before and after percutaneous ethanol injection (PEI, above and below images, respectively). The volume of the nodule before treatment was 4.5 cm3 and after treatment was 1.3 cm3.

All nodules reduced in volume after PEI. In most (73%), the volume of the nodule continued to decrease during long-term follow-up when compared with the short-term follow-up. A complete response to PEI (≥ 70% volume decrease) occurred in 47.2% of the nodules at short-term follow-up and 69.4% of the nodules at long-term follow-up (Figure 1). Complete disappearance of the nodule, reflected by a volume ≤ 0.1 cm^3^, occurred in four nodules (11.1%) at short-term follow-up and seven nodules (19.4%) at long-term follow-up.

**Figure 2:**
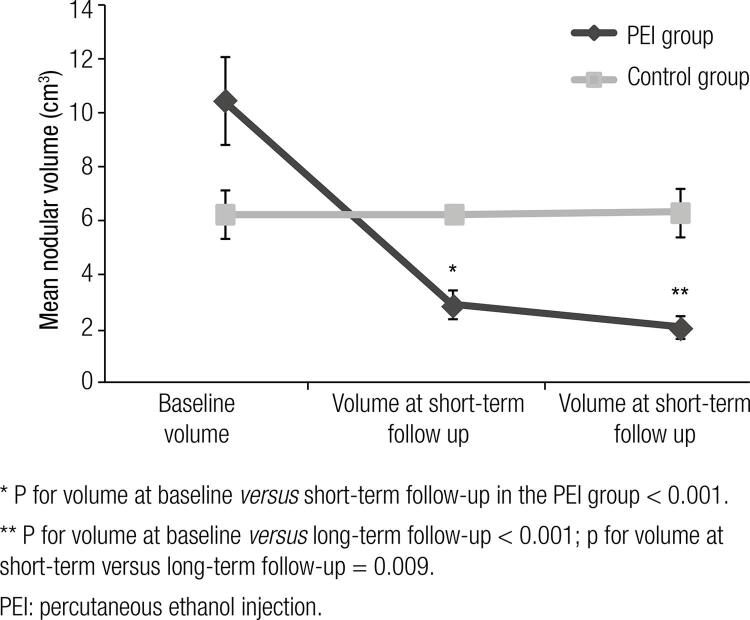
Mean values ± standard error of volumes at baseline, short-term and long-term follow-up in the PEI group and baseline and long-term follow-up in the control group (p for PEI *versus* control groups < 0.001; PEI group, n = 36; control group, n = 13).

In the control group, the initial volume of the nodules ranged from 0.8 to 10.6 cm^3^ (mean 5.8 ± 3.4 cm^3^, 95% CI 3.8–7.9 cm^3^). After a mean follow-up of 26 months, the volumes ranged from 1.7 to 11.8 cm^3^ (mean 6.2 ± 3.0 cm^3^, 95% CI 4.4–8.0 cm^3^, p = 0.507). Comparing the outcomes between both groups, we observe that the nodules in the PEI group showed a significantly larger reduction in volume compared with those in the control group (p < 0.001, Figure 2).

* P for volume at baseline* versus *short-term follow-up in the PEI group < 0.001.

** P for volume at baseline *versus* long-term follow-up < 0.001; p for volume at short-term versus long-term follow-up = 0.009.

PEI: percutaneous ethanol injection.

The characteristics of the different types of nodules in the PEI group are shown in Table 2. Table 3 presents information regarding changes in volume and rates of volume reduction according to the type of nodule. A complete response to PEI at short-term follow-up occurred in 61.5% of the cystic nodules, 62.5% of the predominantly cystic nodules and 26.7% of the predominantly solid nodules (p = 0.106). At long-term follow-up, a complete response to PEI was observed in 69.2% of the cystic nodules, 87.5% of the predominantly cystic nodules and 60% of the predominantly solid nodules (p for comparison between all three groups = 0.359).

**Table 3:** Changes in volume according to the type of nodule after treatment with PEI at short-term and long-term follow-up

		Baseline	Short-term follow-up	Long-term follow-up	p
Cystic (n = 13)	(cm^3^) (%)*	11.6 ± 11.4	2.6 ± 3.2 74.9 ± 22.2	1.9 ± 2.5 80 ± 23.9	0.001
Predominantly cystic (n = 8)	(cm^3^) (%)*	11.6 ± 11.1	3.3 ± 4.7 71.0 ± 19.1	2.2 ± 3.6 85.4 ± 14.0	0.012
Predominantly solid (n = 15)	(cm^3^) (%)*	8.8 ± 8.0	2.9 ± 2.0 59.8 ± 16.2	2.0 ± 1.9 73.5 ± 17.6	< 0.001
Total (n = 36)	(cm^3^) (%)*	10.4 ± 9.8	2.9 ± 3.1 67.7 ± 19.9	2.0 ± 2.5 78.5 ± 19.5	0.001

PEI: percutaneous ethanol injection. * Percentual of nodular reducing.

When we analyzed the outcomes of the cystic and predominantly cystic nodules in combination, we observed a complete response to PEI in 61.9% of these nodules in this combined group at short-term follow-up *versus* 26.7% in the group of predominantly solid nodules (p for comparison of all three groups = 0.037). However, this difference was no longer significant at long-term follow-up, since a complete response was observed in 76.2% in the combined group and 60% in the predominantly solid nodules group (p = 0.465).

Mean baseline serum levels of TSH were 1.56 ± 1.66 mUI/mL and of free T4 were 1.82 ± 1.95 ng/dL. After PEI, mean TSH was 1.33 ± 1.03 mUI/mL and free T4 1.09 ± 0.16 ng/dL. A comparison of the results between baseline and post-PEI showed no statistical difference in TSH (p = 0.879) or free T4 (p = 0.82) levels.

The rate of volume reduction correlated directly with the number of PEI injections/sessions (r = 0.35, p = 0.037) and with the total volume of ethanol injected (r = 0.34, p = 0.045), but not with patient’s age (p = 0.874), baseline nodular volume (p = 0.939) or levels of serum TSH (p = 0.08) and free T4 (p = 0.69).

Most individuals reported mild to moderate pain during injection of ethanol that disappeared at the end of the procedure. One patient developed edema of the sternocleidomastoid muscle immediately after injection, whereas three other patients reported mild pain that lingered for 3 to 4 days. Most patients who reported pain had predominantly solid nodules.

## DISCUSSION

In this study, thyroid nodules treated with PEI showed a mean volume reduction of 78.2%. This outcome was superior to the one observed in the group not treated with this therapy, which on average maintained its volume during follow-up. Studies that focused on other therapeutic options have also shown greater volume reduction of nodules treated with ethanol injection (3,4,15-20). A large series that compared patients with solid thyroid nodules treated with PEI or suppressive doses of levothyroxine (L-T4) showed that PEI was associated with a mean reduction of 47% in nodular volume, compared with a 9% mean reduction with L-T4 (15). In addition to a poorer response, evidence has shown that suppression with L-T4 is associated with side effects and increased cardiovascular morbidity and mortality (3,15,21). Isolate fluid aspiration of cystic and predominantly cystic nodules is also associated with a high rate (60 to 90%) of recurrence, as previously shown (4).

The rate of volume reduction of the nodules in our study was 67.7% one month after PEI and 78.2% after an average of 14 months. These volume reductions are less remarkable than the average reduction of 93% observed in a study (13) with a longer follow-up (7 years) with 110 cystic and predominantly cystic nodules treated with PEI. They demonstrate that at 3 months after PEI, the reduction was 82.6%, showing that the ethanol effect continues to progress over time. However, this study included only cystic and predominantly cystic nodules. Similar results showing progressive volume reduction during long-term follow-up have also been described by other authors. Lee and Ahn (11) observed an even greater reduction in volume during long-term follow-up: 70.1% and 66.1% immediately after PEI and 75.1% and 73.2% after an average of 36 months in solid and cystic nodules respectively. These authors also showed that PEI was effective in both types of nodules, but even though both types eventually achieved similar responses to therapy, the rate of volume reduction and the number of nodules with complete response to treatment were higher among cystic nodules as also shown by others (12,16,17). It is unclear why the effects of ethanol are different immediately after PEI and at long term. Some authors (16) have suggested that the solid part of the nodule may be more resistant to the diffusion of ethanol and other substances. This would explain the greater reduction in volume of cystic nodules immediately after PEI. In addition to that, the aspiration of the fluid before PEI in cystic nodules also contributes to a greater reduction in volume immediately after treatment. However, the sclerosing effect of the ethanol continues to act progressively and at a slow pace, effectively reducing in size nodules with solid content as well. This suggests that the ethanol is continuously absorbed by the solid part of the nodule, resulting in a progressive volume reduction. Another study (11) evaluating the decrease in nodular size with time showed that the reduction at long term is similar in different types of nodules, confirming that solid nodules do not respond as fast as cystic ones to the sclerosing effect of the ethanol.

We did not find difference in serum levels of TSH and free T4 before and after therapy. This corroborates the findings of other authors (5) who evaluated the levels of TSH and free T4 before and 6 months after PEI and did not find a significant difference in serum levels of these hormones, suggesting that PEI does not interfere with the functioning of the gland, in contrast to other types of treatment. Another important study (18) with 125 patients with hyperfunctioning nodules treated with PEI evaluated the response in hormone levels and scintiscan changes after an average of 60 months. In this study, the number of sessions per nodule ranged from 1 to 11 and the amount of injected ethanol per session ranged from 1 to 15.5 mL, which is similar to the amount used in our study (0.4 to 15.5 mL). Results showed that the number of sessions and volume of ethanol were positively associated with the success of the therapy, regardless of the type of nodule. This correlation has been previously shown (19) in a study with cystic nodules treated with 1 to 14 mL of ethanol. In contrast, another study including cystic and solid nodules showed the amount of ethanol injected was associated with greater volume reduction in cystic but not in solid nodules (16).

A recent review comparing PEI and thermal ablation with laser or radiofrequency concluded that in clinical practice, relapsing thyroid cysts are effectively managed with percutaneous ethanol injection treatment, which makes a stronger case to consider PEI as a standard therapy in this circumstance (20). The changes caused by ethanol in thyroid tissue, such as necrosis and fibrosis were considered possible complicating if there is need for further surgery on the gland (22). However, with the IPE technique guided by US, only the nodule suffers action of ethanol, sparing the surrounding tissue, also explaining the low rate of early and late complications (23). Other authors also evaluated macroscopically and microscopically glandular ethanol changes, which claim that the therapy does not affect the extranodular tissue (9). Therefore, treatment with IPE does not preclude the subsequent surgical treatment, which may be performed safely (10).

In conclusion, PEI is an effective therapy for cystic, predominantly cystic and predominantly solid nodules, with sustained long-term efficacy. In addition to that, PEI represents a safe treatment alternative, with minimal, transient and self-limited adverse effects. We propose that this therapy should be considered an important clinical alternative for treatment of benign cystic and mixed thyroid nodules.
